# Facile Preparation of Dense Polysulfone UF Membranes with Enhanced Salt Rejection by Post-Heating

**DOI:** 10.3390/membranes13090759

**Published:** 2023-08-27

**Authors:** Fanxin Kong, Lian You, Dingwen Zhang, Guangdong Sun, Jinfu Chen

**Affiliations:** 1 State Key Laboratory of Heavy Oil Processing, Beijing Key Laboratory of Oil & Gas Pollution Control, China University of Petroleum, Beijing 102249, China18191316601@163.com (D.Z.); jfchen2015@163.com (J.C.); 2 Research Center for Urban & Rural Water Environmental Technology, China Urban and Rural Holding Group Co., Ltd., Beijing 102249, China; 3 Beijing Originwater Membrane Technology Co., Ltd., Beijing 101407, China

**Keywords:** PSf membrane, heat treatment, rejection performance

## Abstract

Polysulfone (PSf) membranes typically have a negligible rejection of salts due to the intrinsic larger pore size and wide pore size distribution. In this work, a facile and scalable heat treatment was proposed to increase the salt rejection. The influence of heat treatment on the structure and performance of PSf membranes was systematically investigated. The average pore size decreased from 9.94 ± 5.5 nm for pristine membranes to 1.18 ± 0.19 nm with the increase in temperature to 50 °C, while the corresponding porosity decreased from 2.07% to 0.13%. Meanwhile, the thickness of the sponge structure decreased from 20.20 to 11.5 μm as the heat treatment temperature increased to 50 °C. The MWCO of PSf decreased from 290,000 Da to 120 Da, whereas the membrane pore size decreased from 5.5 to 0.19 nm. Correspondingly, the water flux decreased from 1545 to 27.24 L·m^−2^·h^−1^, while the rejection ratio increased from 3.1% to 74.0% for Na_2_SO_4_, from 1.3% to 48.2% for MgSO_4_, and from 0.6% to 23.8% for NaCl. Meanwhile, mechanism analysis indicated that the water evaporation in the membranes resulted in the shrinkage of the membrane pores and decrease in the average pore size, thus improving the separation performance. In addition, the desalting performance of the heat-treated membranes for real actual industrial wastewater was improved. This provides a facile and scalable route for PSf membrane applications for enhanced desalination.

## 1. Introduction

Polysulfone (PSf) is one of the typical and promising materials for low-pressure membrane (i.e., microfiltration (MF) and ultrafiltration (UF)) fabrication due to its strong mechanical properties, chemical stability, and heat resistance [1,2,3]. However, the average pore size of commercial PSf membranes is generally larger (higher than 10 nm) than the hydration radius of salt ions (typically less than 0.5 nm), and therefore, PSf membrane has practically no rejection for salt ions [4,5,6].

Heat treatment is a facile and scalable method to adjust the membrane structure and thus dramatically affect the performance of the membrane, which has been universally adopted for membrane fabrication and modification. Su et al. used a two-step heat treatment process to enhance the desalting performance of cellulose acetate hollow fiber membranes (increasing NaCl by 24.61% to 90.17%) with the decrease in average pore size from 0.63 to 0.3 nm [7]. Following a heat treatment at 120 °C, the *d*-spacing of the graphene oxide membrane decreased from 8.35 Å to 7.75 Å, and the selectivity of the ions (Mg^2+^/Ca^2+^, Mg^2+^/Sr^2+^, K^+^/Ca^2+^, and K^+^/Fe^3+^) was substantially improved [8]. Tong et al. investigated the effect of heat treatment temperature on the structure and properties of the Hyflon AD60/PVDF composite hollow fiber membrane, and observed that the water flux initially increased from 7.25 to 10.1 kg m^−2^ h r^−1^ and then decreased to 6 kg m^−2^ h r^−1^ with the increase in heat treatment temperature, whereas the salt rejection ratio exceeded 99% [9]. The permeability of NF membranes with defect-free organosilica separation layers decreased by approximately one order of magnitude, and the salt rejection ratio increased from 90% to 96% with the increase in heat treatment temperature (60–150 °C) [10]. These studies demonstrated that heat treatment can effectively reduce the average pore size and pore size distribution of the membrane, thereby increasing membrane performance. Therefore, it is desirable to systematically verify the feasibility of enhancing the desalination performance of PSf membranes by heat treatment.

Herein, a facile one-step strategy was proposed to improve the salt rejection performance of PSf by heat treatment. The effects of heat treatment temperature on the structure of the PSf membrane were comprehensively investigated using a variety of characterization methods. The pore size distribution and desalination performance of PSf membranes were evaluated by a cross-flow filtration device, and the mechanism for performance improvement was proposed. Finally, the mechanism of the effect of heat treatment on the structure and performance of PSf membranes was proposed.

## 2. Materials and Methods

### 2.1. Materials

Polysulfone UF membrane (PSf) with a molecular weight cut-off (MWCO) of 100,000 Da was purchased from Guochu Technology (Xiamen, China) Co., Ltd. Methanol, and polyethylene glycol and Na_2_SO_4_ were supplied by Macklin Biochemical Technology Co., Ltd. (Shanghai, China). Glycerin was purchased from Shanghai Aladdin Biochemical Technology Co., Ltd., (Shanghai, China). Glycol was purchased from Saen Chemical Technology Co., Ltd. (Shanghai, China). NaCl was purchased from Fuchen Chemical Reagent Co., Ltd. (Tianjin, China). MgSO_4_ was purchased from Tianjin Guangfu Technology Co., Ltd. (Tianjin, China).

### 2.2. Heat Treatment of PSf Membranes and Performance Evaluation

**Figure 1:**
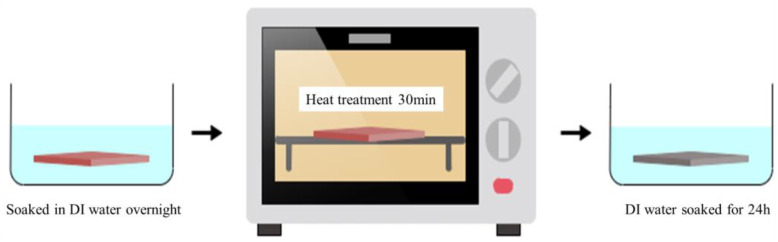
Schematic diagram of PSf membrane undergoing different heat treatments.

The PSf membranes were cut into pieces of 13 × 9 cm^2^ and were soaked in deionized (DI) water overnight in order to completely remove the protection agents from the membrane surface. The membranes were then incubated at different temperatures (20, 30, 40, and 50 °C) for 30 min (Figure 1). These membranes were denoted as PSf*x*, where *x* represented the temperature of the heat treatment. All the fabricated membranes were stored in DI water for at least 24 h before use.

### 2.3. Operational Procedures for Membrane Performance Evaluation

Membrane performance was evaluated by a bench-scale cross-flow filtration system. The effective filtration area was 43 cm^2^. During filtration, the permeate and concentrate were recirculated back to the feed tank. Before each set of tests, the membranes were compacted using DI water at 5.5 bar for 1 h. All the tests were conducted under a filtration pressure and temperature of 5 bar and 25 ± 1 °C, respectively. A cross-flow velocity of 30.4 cm/s was maintained throughout the tests. The rejection tests were carried out using a water matrix containing 1000 mg/L of each inorganic salt (Na_2_SO_4_, MgSO_4_, and NaCl). To further evaluate their potential application, two influents, water from a full-scale RO desalination system at the Yanshan Petrochemical Company (Beijing, China), and full-scale shale-gas-produced water (Weiyuan shale gas Play, Neijiang, China) with a total organic carbon (TOC) content <2.5 mg L^−1^ from an NF membrane, were adopted as low-salinity (total dissolved solids, TDS = 2.58 g L^−1^) and high-salinity (TDS = 15.97 g L^−1^) water, respectively.

The permeate flux (*J_w_*, L m^−2^ h^−1^) and solute rejection ratio (*R*,%) were calculated by using Equations (1) and (2), respectively.

(1) $$J_{w}=\frac{V}{\mathrm{At}}$$

where *A*, *V*, and *t* are the permeating volume (m^3^), membrane area (m^2^), and operation time (h), respectively, and

(2) $$R=(1-\frac{C_{p}}{C_{f}})\times 100\%$$

where $C_{f}$ and $C_{p}$ are the solute concentrations in the feed and permeate, respectively.

### 2.4. Characterization and Analytical Methods

A scanning electron microscope (Hitachi SU8020, Kyoto, Japan) was used for the morphological analysis of the membranes. An Image J software (5/16/2011 11.3.0.0) was adopted to evaluate the pore size (*dp*_avg_) and porosity (*ε*) of the membranes. The surface wettability and water contact angle were measured by using a drop shape analyzer (ShengDing SDC-200S, Dongguan, China). The mechanical properties were tested by using an electronic universal testing machine (Shimadzu, Kyoto, Japan).

During heat treatment, water at the membrane surface and within the membrane matrix was continuously lost. The water loss rate of the membranes was calculated by Equation (3):

(3) $$w=\frac{m_{1}-m_{2}}{m_{1}}$$

where *m*_1_ and *m*_2_ are the mass of the samples before and after water loss (*g*).

The membranes were tested for rejection ratios of neutral probe solutes (glycerol, methanol, ethylene glycol, and PEGs) at 5 bar, and then the experimentally obtained rejection data were fitted using a cumulative distribution function with a log-normal distribution, and the average pore size (*r_p_*) and standard deviation (*S_p_*) were expressed by Equation (4) [11,12,13,14]:

(4) $$f_{R}\left(r\right)=\frac{1}{S_{p}\sqrt{2\pi }}\frac{1}{r}\exp\left(-\frac{{\left(\mathrm{lnr}-{\mathrm{lnr}}_{p}\right)}^{2}}{2S_{p}^{2}}\right)$$

where *r_p_* determines the center of the pore size distribution curves, and *S_p_* determines the sharpness of the pore size distribution curve. *r_p_* and *S_p_* can be estimated by fitting the rejection ratios with log-normal cumulative distribution functions with the obtained rejection ratios of a series of neutral organic molecules. The molecular radius of small neutral organic molecules, i.e., methyl alcohol (32 Da), glycerol (92 Da), xylose (150 Da), glucose (180 Da), and sucrose (342 Da), can be calculated by Equation (5) [13,15]:

(5) $$\log r_{i}=-1.4962+0.4654\log\left(\mathrm{MW}\right)$$

The molecular radius of PEGs (MW of 1000, 6000, 10,000, and 100,000 Da) can be obtained by Equation (6) [13,15]:

(6) $$r_{i}=16.73\times {\mathrm{MW}}^{0.557}\times 10^{-3}$$

where the *r_i_* (nm) is the molecular radius.

TDS and the concentration of probe solutes were measured by a conductivity meter (FE38, Mettler Toledo, Greifensee, Switzerland) and a TOC analyzer (TOC-VCPH, Shimadzu Corp., Kyoto, Japan), respectively. The specific anions and cations were measured using an ion chromatograph (CH-9100, Metrohm AG, Herisau, Switzerland) equipped with Metrosep A Supp 5-250/4.0 (Metrohm AG, Herisau, Switzerland) (250 × 4.0 mm, and 5 μm) and Metrosep C 6-150/4.0 (Metrohm AG, Herisau, Switzerland) (4.0 × 150 mm, and 5 μm) columns, respectively.

## 3. Results and Discussion

### 3.1. Characterizations of PSf Membranes

**Figure 2:**
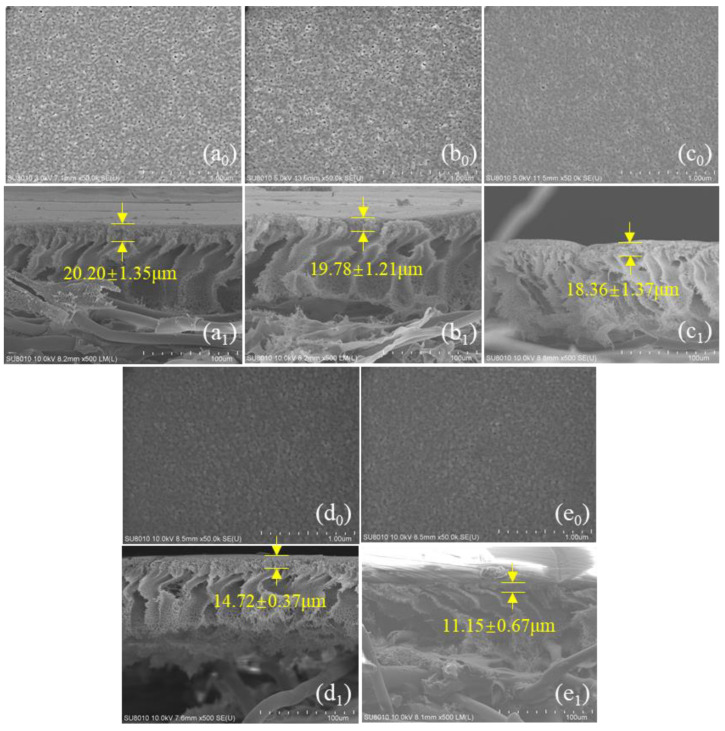
Surface morphology of PSf_untreated_ (**a_0_**), PSf_20_ (**b_0_**), PSf_30_ (**c_0_**), PSf_40_ (**d_0_**), and PSf_50_ (**e_0_**); cross-section morphology of PSf_untreated_ (**a_1_**), PSf_20_ (**b_1_**), PSf_30_ (**c_1_**), PSf_40_ (**d_1_**), and PSf_50_ (**e_1_**).

**Figure 3:**
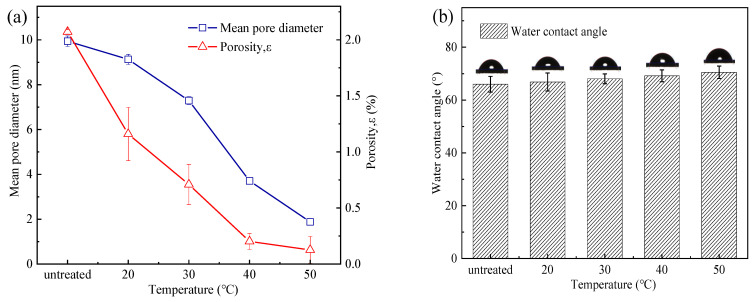

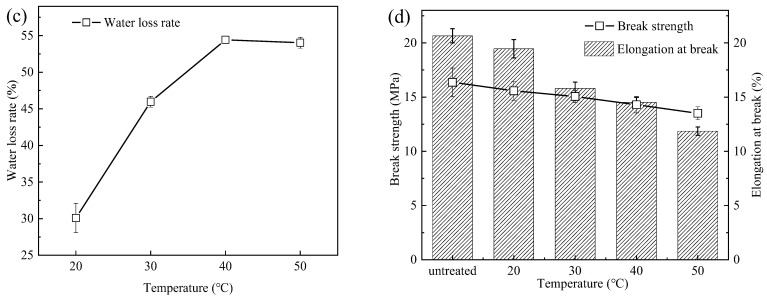
(**a**) Average pore size and porosity, (**b**) water contact angle, (**c**) water loss rate, and (**d**) mechanical properties of PSf membranes at different heat treatment temperatures.

The surface of the untreated PSf membranes was evenly distributed with various pore sizes (Figure 2(a0)). The density of the membrane pores gradually diminished, and the morphology of the pores became progressively blurred with the increase in heat treatment temperature. It was obvious that the morphology of the pores on the membrane surface became blurred when the heat treatment temperature exceeded 40 °C (Figure 2(d0)). In addition, the average pore size *(dp*_avg_) and porosity (*ε*) of the membranes were quantitatively evaluated by the Image J software (Figure 3a). In general, the average pore size and porosity of the membranes gradually decreased as the temperature of the heat treatment increased. The average pore size of the membranes decreased from 9.94 nm (PSf_untreated_) to 1.18 nm (PSf_50_), and the corresponding porosity decreased from 2.07% to 0.13% as the temperature increased. The increase in temperature of the heat treatment caused the membrane pores to shrink. In addition, cross-section analysis (Figure 2(a1)) of the PSf membranes indicated an asymmetrical structure consisting of a fingerlike porous sub-layer and a spongy top layer. Meanwhile, the thickness of the spongy structure decreased from 20.20 to 11.5 μm with the heat treatment temperature increased to 50 °C. A similar phenomenon was observed that PES membranes shrank by 18% and 40% post-treatment at 150 °C and 180 °C, respectively [16].

The FTIR-ATR spectra of the PES membranes before and after heat treatment were similar [16,17], proving that the water loss of the membranes was a physical change. As a result, there was almost no difference in the contact angle for the membranes (Figure 3b) between that of the PSf_untreated_ and that of the PSf treated under different temperatures. The water loss rate of the PSf membranes increased from 0 to 54% as the temperature increased from 0 to 50 °C (Figure 3c). The increase in temperature accelerated the evaporation of water, resulting in the loss of free water in the membrane surface and matrix [18]. When the temperature exceeded 40 °C, the water loss rate marginally changed.

The mechanical properties of the membranes were analyzed by the break strength and elongation at break (Figure 3d). As the temperature increased from 0 to 50 °C, the break strength of the PSf membranes decreased from 16.4 MPa to 13.5 MPa, and the elongation at break decreased from 20.7% to 11.9%. As the treatment temperature increased, the water loss of the membranes gradually increased, and the elongation at break decreased. This was consistent with the phenomenon observed by Xu [19] and Yuan [20] et al. When the temperature increased from 25 °C to 45 °C, the break strength of the PSf hollow fiber membranes changed from 431.1 cN to 430.3 cN, and the elongation at break changed from 41.6% to 20.7%. The elongation at break of PVDF-PFSA membranes decreased from 61.4% to 42.0% when the temperature ranged from 40 °C to 90 °C.

### 3.2. Performance of PSf Membranes

#### 3.2.1. MWCO and Pore Size Distribution

**Figure 4:**
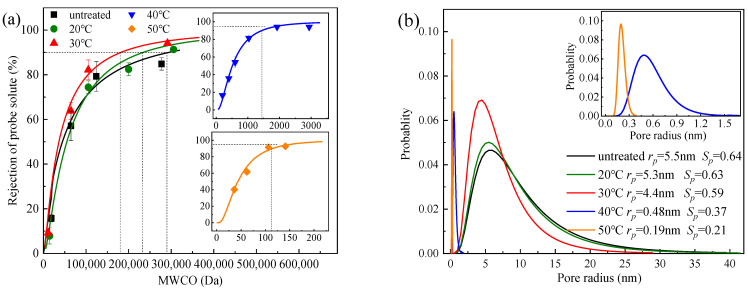
(**a**) MWCO of PSf membranes with different heat treatment temperatures; (**b**) pore size distribution of PSf membranes after heat treatment at different temperatures.

The rejection of neutral probe solutes with different molecular weights (i.e., methanol, ethylene glycol, glycerol, polyethylene glycol, and PEGs) was mainly due to the steric hindrance effect [21], which could be used to analyze the mean pore size and pore size distribution of the membranes. The MWCO of the PSf_untreated_ membranes was approximately 290,000 Da, slightly higher than that of 100,000 Da from the supplier, probably due to the difference in test conditions [22]. The MWCO of the membranes decreased from 290,000 Da to 120 Da with the temperature increased from 0 to 50 °C (Figure 4a). The corresponding average pore size of PSf_50_ (*r_p_* = 0.19 nm, and *S_p_* = 0.21) was one order of magnitude lower than that of PSf_untreated_ (*r_p_* = 5.5 nm, and *S_p_* = 0.64), which was well consistent with the shrinkage of the membrane pores and the reduction in porosity observed in the SEM image (Figure 3a).

#### 3.2.2. Slat Rejection

**Figure 5:**
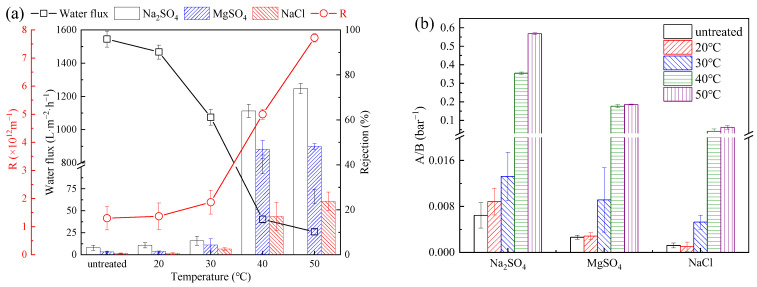
(**a**) Pure water flux, separation performance, and mass transfer resistance of PSf membranes for different salt solutions under different heat treatments; (**b**) water/Na_2_SO_4_ selectivity, water/MgSO_4_ selectivity, and water/NaCl selectivity (A/B) of PSf membranes under different heat treatments.

The water flux and performance of the PSf membranes were tested and evaluated at 5 bar (Figure 5a). The water flux of the PSf membranes decreased from 1545 to 27.24 L·m^−2^·h^−1^ as the heat treatment temperature increased to 50 °C. The rejection ratio increased from 3.1% to 74.0% for Na_2_SO_4_, from 1.3% to 48.2% for MgSO_4_, and from 0.6% to 23.8% for NaCl. The water/salt selectivity of the PSf membranes was calculated (Figure 5b) [23], and the water/salt selectivity of the membranes increased significantly as the heat treatment temperature increased to 40 °C (water/Na_2_SO_4_ selectivity to 0.35 bar^−1^, water/MgSO_4_ selectivity to 0.18 bar^−1^, and water/NaCl selectivity to 0.04 bar^−1^). The decrease in water flux and the increase in the rejection ratios were mainly from the decreased pore size of the PSf membranes. The temperature increase led to the shrinkage of the membrane pores and slight collapse of the membrane structure (Figure 2) [24]. Increased mass transfer resistance of the membranes resulted in a sharp decrease in water flux. According to the resistance-in-series model, the mass transfer resistance (R) was calculated to have tremendously (Figure 5a) increased from 1.30 × 10^12^ m^−1^ (PSf_untreated_) to 7.72 × 10^13^ m^−1^ (PSf_50_) [25].

In addition, salt rejection was mainly governed by the steric hindrance and electrostatic effects. Ion rejection was exclusively governed by the size exclusion (steric hindrance) mechanism due to the neutral properties of the PSf membranes [26]. Therefore, the decrease in membrane pore size was supposed to effectively improve the rejection performance due to the steric hindrance. In comparison, commercial NF270 membranes had an average pore size of 0.365 nm [13], which was higher than that of the PSf_50_ (0.19 nm). However, the rejection ratio of Na_2_SO_4_ for NF270 was higher than 95% due to the electrostatic effect of a negatively charged surface [27]. Therefore, the Na_2_SO_4_ rejection ratio can be further enhanced by surface modification or grafting charged functional groups, which should be further studied in the future.

### 3.3. Desalinization Performance of Industrial Wastewater

The ion rejection performance of the real water matrix differed from that of the single salts, especially for ions with a small hydrated radius [10,28]. For this purpose, it was important to evaluate the desalinization performance of actual wastewater for the potential application of the PSf membrane. The desalinization performance of the wastewater (petrochemical wastewater and shale-gas-produced water) with different salinities was investigated using PSf_untreated_ and PSf_50_ at 5 bar.

**Figure 6:**
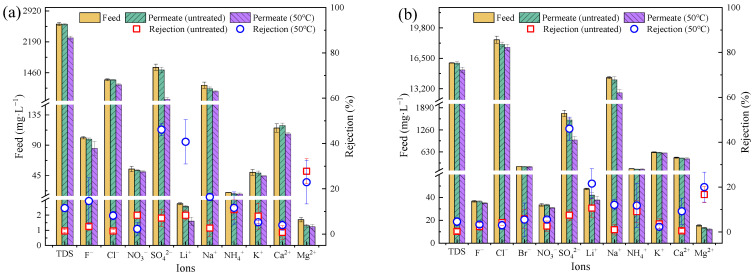
Desalination performance of PSf*_untreated_* membranes and PSf_50_ membranes for (**a**) petrochemical wastewater and (**b**) shale-gas-produced water.

For petrochemical wastewater (Figure 6a), the PSf_untreated_ had a marginal rejection ratio of ions (<10%). There was no rejection of various types of ions due to the average pore size of PSf_untreated_ (5.5 nm) being much larger than the hydrated radius of the ions. After treatment at 50 °C, the average pore size of PSf_50_ was 0.19 nm, but the pore size distribution was still wide (*S_p_* = 0.21) (Figure 4b). PSf_50_ showed a significant increase in the rejection of both SO_4_^2−^ (from 7.1% to 46.1%) and Li^+^ (from 6.7% to 40.7%), but a slight increase in the rejection of Cl^−^ (from 0.6% to 8.2%) and Na^+^ (from 2.6% to 16.4%).

For high-salinity shale-gas-produced water (Figure 6b), the ion rejection ratios for PSf_untreated_ and PSf_50_ were similar to that of petrochemical wastewater. Notably, the removal of SO_4_^2−^ by PSf_50_ in the actual wastewater was only 46%, which was slightly lower than that of the single salts (74.0% for Na_2_SO_4_ and 48.2% for MgSO_4_). The TDS concentration of the wastewater was much higher than the single salt concentration, leading to large differences in desalination performance [29,30].

In general, the removal of SO_4_^2−^ by the PSf_50_ was approximately 46% for both the shale-gas-produced water and the petrochemical wastewater, which was much higher than that of the pristine membranes. However, the desalination performance was unsatisfactory. Other strategies (i.e., surface modification or surface grafting) [31,32,33] might could be a possible potential approach to enhance the electrostatic effect of the treated PSf membrane, which should be further studied and investigated in the future.

### 3.4. Implication of Heating on the Structure and Performance of the PSf Membrane

**Figure 7:**
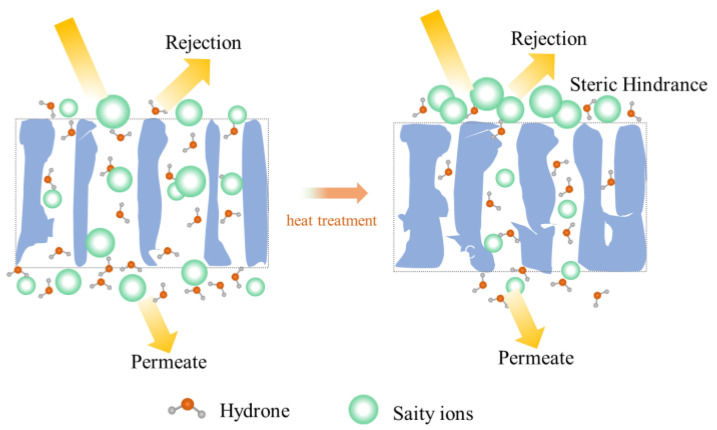
Heat treatment mechanism of PSf membranes.

A schematic diagram of the heat treatment of the PSf structure was proposed (Figure 7). The average pore size of the untreated PSf membranes was larger than the hydration radius of various salt ions (Figure 4b) [6]. The evaporation of water molecules occurred during heat treatment and caused the slight collapse of the membrane structure. Tensile stresses existed between the polymers due to hydrogen bonding between water molecules [24,34]. This resulted in varying degrees of shrinkage of the membrane pores. Therefore, the membrane pore size reduced, and the mass transfer resistance increased. Based on the size exclusion (steric hindrance) mechanism [26], the desalination performance of the PSf membranes was enhanced (Figure 5). Consequently, heat treatment was proved to be a facile and effective method to improve the desalination performance of PSf membranes.

## 4. Conclusions

In this study, the pore size distribution of PSf membranes was tailored by the heat treatment of PSf membranes at different temperatures to improve the desalination rate of the PSf membranes. As the temperature increased to 50 °C, the average pore size of the PSf membranes decreased from 9.94 (5.5) nm to 1.18 (0.19) nm, and the porosity decreased from 2.07% to 0.13%. The MWCO decreased from 290,000 Da to 120 Da, and the pore size distribution decreased from 0.64 to 0.21, while the temperature increased from 0 to 50 °C. Correspondingly, the water flux decreased from 1545 to 27.24 L·m^−2^·h^−1^, and the rejection ratios of Na_2_SO_4_, MgSO_4_, and NaCl increased from 3.1% to 74.0%, from 1.3% to 48.2%, and from 0.6% to 23.8%, respectively. Mechanistically, the shrinkage of membrane pores during the heating process resulted in the increase in the salt rejection ratios. Therefore, the shrinkage of the PSf membrane pores can be tailored by adjusting the heat treatment conditions, and thus, the desalination performance of the membrane could be substantially improved. Additionally, the desalting performance of the heat-treated membranes for actual industrial wastewater (both high and low salinity) was improved. This work provides favorable support for future applications of UF membranes for partial desalination.

## Data Availability

Not applicable.
